# Supplementary material for: Multiple global radiations in tadpole shrimps challenge the concept of ‘living fossils’
Source: PeerJ. 2013 Apr 2;1:e62. doi: 10.7717/peerj.62 (PMC3628881; doi:10.7717/peerj.62)
Supplement: Table S1 — Acession numbers within each ML cluster defined by the GMYC model. Accessions in bold were used in the multilocus phylogenetic analysis. [file peerj-01-62-s003.docx]

| **Cluster ID** | **Accession** |
| --- | --- |
| *L. apus* | DQ834543.1 |
|  | DQ834544.1 |
|  | DQ148285.1 |
|  | EF189669.1 |
|  | **JX110638** |
| *L. arcticus* | HM425362.1 BOLD |
|  | AF209067.1 |
|  | DQ834545.1 |
|  | HM425363.1 BOLD |
|  | HM425361.1 BOLD |
|  | DQ148286.1 |
|  | HM425364.1 BOLD |
|  | HM425365.1 BOLD |
|  | **JX110641** |
| *L. cf. couesii* (Italy) | DQ148290.1 |
|  | DQ148288.1 |
|  | DQ834549.1 |
|  | **DQ834546.1** |
|  | DQ148289.1 |
|  | DQ834547.1 |
|  | DQ834548.1 |
|  | DQ834550.1 |
|  | DQ148287.1 |
| *L. couesii* | **DQ310622.1** |
|  | DQ889156.1 |
| *L. lemmoni* | **GQ144447.1** |
| *L. lubbocki* | DQ148284.1 |
|  | DQ834542.1 |
|  | **JX110643** |
|  | DQ148283.1 |
|  | DQ148282.1 |
|  | DQ834541.1 |
|  | DQ834540.1 |
| *L. packardi* | **JX110642** |
| *L. viridis* | **JN175225**  JN175226  JN175227  JN175228  JN175229 |
| *L. cf. couesii* (Sardinia) | **JX110640** |
| *T. baeticus* | FN691434.1 |
|  | **JX110645** |
|  | FN691433.1 |
| *T. cancriformis* | EF675884.1 |
|  | EF675896.1 |
|  | EF675892.1 |
|  | EF675885.1 |
|  | EF675895.1 |
|  | EF675887.1 |
|  | EF675888.1 |
|  | EF675893.1 |
|  | EF675886.1 |
|  | EF675891.1 |
|  | EF675894.1 |
|  | EF675897.1 |
|  | EF675889.1 |
|  | EF675890.1 |
|  | FN691430.1 |
|  | EF675899.1 |
|  | EF675898.1 |
|  | GQ328960.1 |
|  | GQ144445.1 |
|  | FN691431.1 |
|  | DQ369312.1 |
|  | EF675859.1 |
|  | EF675860.1 |
|  | EF675853.1 |
|  | EF675832.1 |
|  | EF675842.1 |
|  | EF675843.1 |
|  | EF675844.1 |
|  | EF675850.1 |
|  | EF675845.1 |
|  | EF675831.1 |
|  | EF675836.1 |
|  | EF675835.1 |
|  | EF675879.1 |
|  | EF675851.1 |
|  | EF675829.1 |
|  | EF675849.1 |
|  | EF675838.1 |
|  | EF675837.1 |
|  | EF675840.1 |
|  | EF675861.1 |
|  | EF675857.1 |
|  | EF675830.1 |
|  | EF675848.1 |
|  | EF675854.1 |
|  | EF675856.1 |
|  | EF675852.1 |
|  | EF675834.1 |
|  | EF675846.1 |
|  | EF675839.1 |
|  | EF675833.1 |
|  | EF675847.1 |
|  | EF675841.1 |
|  | EF675858.1 |
|  | EF675855.1 |
|  | DQ369315.1 |
|  | **JX110644** |
|  | EF675869.1 |
|  | EF675872.1 |
|  | EF675866.1 |
|  | EF675868.1 |
|  | EF675878.1 |
|  | DQ369317.1 |
|  | EF675874.1 |
|  | EF675875.1 |
|  | EF675870.1 |
|  | EF675864.1 |
|  | EF675867.1 |
|  | EF675873.1 |
|  | EF675863.1 |
|  | EF675871.1 |
|  | EF675876.1 |
|  | EF675865.1 |
|  | EF675877.1 |
|  | EF675862.1 |
|  | DQ369314.1 |
|  | EF675827.1 |
|  | DQ369313.1 |
|  | FN691432.1 |
|  | EF675826.1 |
|  | NC 004465.1 |
|  | EF675828.1 |
|  | EF189678.1 |
|  | AB084514.1 |
|  | DQ148291.1 |
|  | DQ664196.1 |
|  | EF675880.1 |
|  | EF675881.1 |
|  | DQ369316.1 |
|  | EF675882.1 |
|  | EF675883.1  JN175241.1  JN175234.1\| |
| *T. cf. australiensis* sp. 1 | **EF189677.1** |
| *T. cf. australiensis* sp. 2 | **DQ310624.1** |
|  | DQ889135.1 |
| *T. cf. australiensis* sp. 3 | **DQ310625.1** |
| *T. cf. australiensis* sp. A | **DQ343234.1** |
| *T. cf. australiensis* sp. B | **DQ343235.1** |
| *T. cf. australiensis* (Lake Carey) | **JN175235.1**  JN175236.1 |
| *Triops cf australiensis* (Baladonia Rock) | **JN175233.1**  JN175237.1  JN175244.1 |
| *T. cf australiensis* (Ayers Rock) | **JN175245.1** |
| *T. cf australiensis* (Paynes Find) | **JN175242.1** |
| *T. cf australiensis* (Gibb Rock) | **JN190396.1**  JN190398.1 |
| *T. cf australiensis* (Walga Rock) | **JN175238.1**  JN175232.1  JN175230.1  JN175231.1  JN175240.1  JN175239.1  JN190397.1  JN175238.1 |
| *T. cf. longicaudatus* sp. 1 | HQ908557.1 |
|  | **HQ908544.1** |
|  | HQ908563.1 |
|  | HQ908559.1 |
|  | HQ908554.1 |
|  | HQ908552.1 |
|  | HQ908548.1 |
|  | HQ908547.1 |
|  | HQ908550.1 |
|  | HQ908567.1 |
|  | HQ908564.1 |
|  | HQ908551.1 |
|  | HQ908556.1 |
|  | HQ908546.1 |
|  | HQ908565.1 |
|  | HQ908558.1 |
|  | HQ908561.1 |
|  | HQ908549.1 |
|  | HQ908566.1 |
|  | HQ908555.1 |
|  | HQ908560.1 |
|  | HQ908553.1 |
|  | HQ908545.1 |
|  | HQ908562.1 |
| *T. emeritensis* | EF675900.1 |
|  | **FN691435.1** |
| *T. cf. granarius* (Japan) | GQ144446.1 |
|  | **JX110646** |
| *T. cf. granarius* (Namibia) | **JX110639** |
| *T. cf. granarius* (South Africa) | **JN175223.1**  JN175224.1 |
| *T. cf. longicaudatus* sp. 2 | HQ908538.1 |
|  | HQ908539.1 |
|  | HQ908526.1 |
|  | HQ908523.1 |
|  | HQ908542.1 |
|  | HQ908531.1 |
|  | HQ908524.1 |
|  | HQ908530.1 |
|  | **JX110649** |
|  | HQ908519.1 |
|  | HQ908527.1 |
|  | HQ908525.1 |
|  | HQ908521.1 |
|  | HQ908534.1 |
|  | HQ908540.1 |
|  | HQ908541.1 |
|  | HQ908518.1 |
|  | HQ908522.1 |
|  | HQ908543.1 |
|  | HQ908537.1 |
|  | HQ908536.1 |
|  | HQ908533.1 |
|  | HQ908535.1 |
|  | HQ908529.1 |
|  | HQ908520.1 |
|  | HQ908517.1 |
|  | HQ908532.1 |
|  | HQ908528.1 |
|  | GU475465.1 |
|  | HM883938.1 BOLD |
|  | HM883939.1 BOLD |
|  | HM883941.1 BOLD |
|  | HM883940.1 BOLD |
|  | HM883942.1 BOLD |
|  | GQ144444.1 |
|  | DQ310623.1 |
|  | JX110647 |
| *T. mauritanicus* | EF675905.1 |
|  | EF675904.1 |
|  | EF675901.1 |
|  | EF675903.1 |
|  | EF675902.1 |
|  | **FN691439.1** |
|  | FN691440.1 |
|  | FN691443.1 |
|  | FN691442.1 |
|  | FN691441.1 |
| *T. newberryi* | HQ908510.1 |
|  | HQ908508.1 |
|  | HQ908509.1 |
|  | HQ908496.1 |
|  | HQ908507.1 |
|  | HQ908499.1 |
|  | HQ908501.1 |
|  | HQ908502.1 |
|  | HQ908512.1 |
|  | HQ908500.1 |
|  | HQ908498.1 |
|  | HQ908504.1 |
|  | HQ908505.1 |
|  | HQ908506.1 |
|  | HQ908503.1 |
|  | HQ908511.1 |
|  | HQ908513.1 |
|  | HQ908516.1 |
|  | HQ908514.1 |
|  | HQ908497.1 |
|  | **JX110648** |
|  | HQ908515.1 |
|  | NC 006079.1 |
|  | AY639934.1  JN175243.1 |
| *T. simplex* | FN691438.1 |
|  | **FN691436.1** |
|  | FN691437.1 |
| *T. cf. mauritanicus* (E. Spain) | EF675906.1 |
|  | **EF675907.1** |
|  | EF675908.1 |
| *T. cf. granarius* (Russia) | **EF521890.1** |
| *T. vicentinus* | **FN691444.1** |
