# Supplementary material for: Multiple global radiations in tadpole shrimps challenge the concept of ‘living fossils’
Source: PeerJ. 2013 Apr 2;1:e62. doi: 10.7717/peerj.62 (PMC3628881; doi:10.7717/peerj.62)
Supplement: Table S2 [file peerj-01-62-s004.docx]

| **STU** | **12S** | **16S** | **COI** | **28S** | **EF1** | **RNA pol** | **Glyc synth** |
| --- | --- | --- | --- | --- | --- | --- | --- |
| *T. baeticus* | KC466334 | KC466343 | JX110645 |  | KC466360 | KC466372 |  |
| *T. cancriformis* | KC466333 | KC466342 | JX110644 | KC466348 | KC466359 | KC466368 | KC466363 |
| *T. cf. australiensis* sp. 1 |  | EF189616 | EF189677 | EF189662 | EF189595 |  |  |
| *T. cf. australiensis* sp. 2 |  |  | DQ310624 |  |  |  |  |
| *T. cf. australiensis* sp. 3 |  |  | DQ310625 |  |  |  |  |
| *T. cf. australiensis* sp. A | DQ343232 |  | DQ343234 |  |  |  |  |
| *T. cf. australiensis* (Lake Carey) | JN175250 |  | JN175235 |  |  |  |  |
| *Triops cf australiensis* (Baladonia Rock) | JN175259 |  | JN175233 |  |  |  |  |
| *T. cf australiensis* (Ayers Rock) | JN175263 |  | JN175245 |  |  |  |  |
| *T. cf australiensis* (Paynes Find) | JN175254 |  | JN175242 |  |  |  |  |
| *T. cf australiensis* (Gibb Rock) | JN175265 |  | JN190396 |  |  |  |  |
| *T. cf australiensis* (Walga Rock) | JN175253 |  | JN175238 |  |  |  |  |
| *T. cf. australiensis* sp. B | DQ343233 |  | DQ343235 |  |  |  |  |
| *T. cf. granarius* (Japan) | KC466335 | KC466341 | JX110646 | KC466347 | KC466358 | KC466369 |  |
| *T. cf. granarius* (Namibia) | AM269423 | AM269433 | JX110639 | AM269444 |  |  |  |
| *T. cf. granarius* (Tunisia) | AM269421 | AM269431 |  | AM269442 |  |  |  |
| *T. cf. granarius* (South Africa) | JN175248 |  | JN175223 |  |  |  |  |
| *T. cf. longicaudatus* sp. 1 |  |  | HQ908544 |  |  |  |  |
| *T. cf. longicaudatus* sp. 2 | KC466336 | KC466344 | JX110649 |  | KC466356 | KC466371 |  |
| *T. emeritensis* | FN691428 | AM183882 | FN691435 |  |  |  |  |
| *T. gadensis* | FN691421 | FN689863 |  |  |  |  |  |
| *T. mauritanicus* | AM184177 | AM183873 | FN691439 |  |  |  |  |
| *T. newberryi* | KC466337 | KC466345 | JX110648 | KC466346 | KC466357 | KC466370 | KC466364 |
| *T. simplex* | AM184172 | AM183867 | FN691436 |  |  |  |  |
| *T. cf. mauritanicus* (E. Spain) |  |  | EF675907 |  |  |  |  |
| *T. cf. granarius* (Russia) |  |  | EF521890 |  |  |  |  |
| *T. vicentinus* | FN691426 | FN689867 | FN691444 |  |  |  |  |
| *L. apus* | AF494483 | DQ148279 | JX110638 | KC466349 | AF526293 |  |  |
| *L. arcticus* | AY159569 / AJ583699 | DQ834538 | JX110641 | AF209047 | KC466353 | KC466365 | KC466361 |
| *L. bilobatus* | AJ000828 |  |  |  |  |  |  |
| *L. couesii* (Canada) | AJ000827 |  | DQ310622 |  |  |  |  |
| *L. cf. couesii* (Apulia) | DQ148274 | DQ148280 | DQ834546 |  |  |  |  |
| *L. cf. couesii* (Sardinia) | KC466331 | KC466339 | JX110640 | KC466351 | KC466355 | KC466366 | KC466362 |
| *L. cryptus* | AJ000824 |  |  |  |  |  |  |
| *L. lemmoni* | AY115604 | AY115614 | GQ144447 |  |  |  |  |
| *L. lubbocki* | KC466332 | KC466340 | JX110643 | KC466350 |  | KC466367 |  |
| *L. packardi* | KC466330 | KC466338 | JX110642 | KC466352 | KC466354 |  |  |
| *L. viridis* | JN175246 |  | JN175225 |  |  |  |  |
| *Artemia* sp. | X69067 | FJ007834 | DQ401269 | AY210805 | GQ122208 | U10331* |  |
| *Streptocephalus seali* |  | JX439913 | AY519832 |  | AY305480 | AY305628 | GQ88703 |
| *Daphnia magna* | JN903683 | GQ343288 | EU702133 | AF532883 | AB734039 |  | GQ887666 |
| *Daphnia pulex* | JN903685 | JN874607 | HM622593 | AY630618 | EFX85268 | EFX75312 | EFX74238 |
| *Eulimnadia* sp. | AY779680 | EF189604 | FJ499139 | FJ499231 | FJ499103 |  |  |
| *Limnadia lenticularis* | AF494471 | EF189609 | FJ499183 | FJ499284 | AF063412 | AF138989 | GQ887703 |
| *Lynceus* sp. | AF494479 | EF189612 | HQ966453 | EF189653 | AF526294 | AY305581.1 | GQ887686 |
