# Supplementary material for: Multiple global radiations in tadpole shrimps challenge the concept of ‘living fossils’
Source: PeerJ. 2013 Apr 2;1:e62. doi: 10.7717/peerj.62 (PMC3628881; doi:10.7717/peerj.62)
Supplement: Table S3 [file peerj-01-62-s005.docx]

| **Partition** | **Partition contents** | **Substitution model** |
| --- | --- | --- |
| 1 | 12S | GTR +G |
| 2 | 16S | GTR +G |
| 3 | 28S | GTR +G |
| 4 | COI 1^st^ position | GTR +G |
| 5 | COI 2^nd^ position, EF1 2^nd^ position, Glyc. Synth. 2^nd^ position, RNA P. II 2^nd^ position | GTR +G |
| 6 | COI 3^rd^ position | GTR +G |
| 7 | EF1 1^st^ position, Glyc. Synth. 1^st^ position, RNA P. II 1^st^ position | GTR +G |
| 8 | EF1 3^rd^ position | GTR +G |
| 9 | Glyc. Synth. 3^rd^ position, RNA P. II 3^rd^ position | GTR +G |
