# Supplementary material for: Multiple global radiations in tadpole shrimps challenge the concept of ‘living fossils’
Source: PeerJ. 2013 Apr 2;1:e62. doi: 10.7717/peerj.62 (PMC3628881; doi:10.7717/peerj.62)
Supplement: Table S5 — Only Nostostraca and one outgroup were included in the analysis. Analysis conducted using LASER. The best scoring model is shaded. [file peerj-01-62-s007.docx]

| **Model** | **Parameters** | **Model type** | **LH** | **r1** | **r2** | **r3** | **a** | **xp** | **k** | **st 1** | **st 2** | **AIC** | **dAIC** |
| --- | --- | --- | --- | --- | --- | --- | --- | --- | --- | --- | --- | --- | --- |
| pureBirth | 1 | RC | -81.70 | 0.0185 | NA | NA | NA | NA | NA | NA | NA | 165.40 | 23.95 |
| bd | 2 | RC | -73.02 | 0.0000 | NA | NA | 0.9999 | NA | NA | NA | NA | 150.04 | 8.59 |
| DDX | 2 | RV | -74.37 | 0.0044 | NA | NA | NA | -0.5862 | NA | NA | NA | 152.74 | 11.29 |
| DDL | 2 | RV | -81.70 | 0.0185 | NA | NA | NA | NA | 645575 | NA | NA | 167.40 | 25.95 |
| yule2rate | 3 | RV | -70.34 | 0.0035 | 0.0299 | NA | NA | NA | NA | 73.28 | NA | 146.68 | 5.23 |
| yule3rate | 5 | RV | -65.72 | 0.0035 | 0.0365 | 0.0043 | NA | NA | NA | 73.28 | 6.00 | 141.45 | 0.00 |
